# Conservation concerns associated with low genetic diversity for K’gari–Fraser Island dingoes

**DOI:** 10.1038/s41598-021-89056-z

**Published:** 2021-05-04

**Authors:** G. C. Conroy, R. W. Lamont, L. Bridges, D. Stephens, A. Wardell-Johnson, S. M. Ogbourne

**Affiliations:** 1 Genecology Research Centre, University of the Sunshine Coast, Maroochydore DC, QLD 4558 Australia; 2 School of Science, Technology and Engineering, University of the Sunshine Coast, Maroochydore DC, QLD 4558 Australia; 3 Zoological Genetics, Inglewood, Adelaide, SA 5133 Australia; 4 Senior Professional Fellow, Curtin University, Bentley, WA Australia

**Keywords:** Genetic variation, Conservation biology, Ecological genetics

## Abstract

The dingo population on world heritage-listed K’gari-Fraser Island (K’gari) is amongst the most well-known in Australia. However, an absence of population genetic data limits capacity for informed conservation management. We used 9 microsatellite loci to compare the levels of genetic diversity and genetic structure of 175 K’gari dingo tissue samples with 264 samples from adjacent mainland regions. Our results demonstrated that the K'gari population has significantly lower genetic diversity than mainland dingoes (*A*_*R*_, *H*_*E*_, *P*_*AR*_; *p* < 0.05) with a fourfold reduction in effective population size (*N*_*e*_ = 25.7 vs 103.8). There is also strong evidence of genetic differentiation between the island and mainland populations. These results are in accordance with genetic theory for small, isolated, island populations, and most likely the result of low initial diversity and founder effects such as bottlenecks leading to decreased diversity and drift. As the first study to incorporate a large sample set of K’gari dingoes, this provides invaluable baseline data for future research, which should incorporate genetic and demographic monitoring to ensure long-term persistence. Given that human-associated activities will continue to result in dingo mortality, it is critical that genetic factors are considered in conservation management decisions to avoid deleterious consequences for this iconic dingo population.

## Introduction

The dingo (*Canis dingo*^1,2^*)* is a highly adaptable canid synonymous with the Australian landscape, and occurs in all habitat types, including subtropical areas, alpine regions and across arid inland Australia^3^. The taxonomy of the dingo remains under debate, and historical nomenclature has included *C. familiaris dingo* Blumenbach, 1780; *C. antarticus* Kerr, 1792; and *C. dingo* Meyer, 1793^2,4^. Contemporary taxonomic arguments exist for both *C. dingo *^1,2,5^ and *C. familiaris *^4,6^, and although considerable debate also remains regarding the timing of their arrival to Australia, we adopt *C. dingo* in this paper due to the unique evolutionary history of the dingo^2,7–10^. Archeological evidence of the dingo’s presence in Australia only extends to 3500 years^11^ whilst genetic evidence ranges from 5000^10,12^ to 18,000 years ago^13^**.** The latter figure is considered unlikely, given their historical and ongoing absence from Tasmania, which has been geographically isolated from mainland Australia for ~ 12,000 years^14,15^.

While the initial geographic radiation of dingoes remains contested, various authors report introduction via a single, small founder event from SE Asia^10,16^. However, the existence of unique mitochondrial haplotypes amongst NE and NW Australian populations, suggests greater phylogenetic complexity, and the possibility of multiple founder events^13^. Although dingoes are listed as threatened in Victoria and protected as native wildlife in the Australian Capital Territory (ACT), New South Wales (NSW) and Victoria, they are also declared pests in all Australian states and territories. In Queensland, they are protected under the *Nature Conservation Act 1992* in conservation areas such as national parks but listed as a declared pest under the *Rural Lands Protection Act 1985*, with an impetus for landowners to cull them under the *Biosecurity Act 2015*^17^*.* This contradictory legal status results in the dingo being protected in some areas (national parks, Aboriginal reserves), but subject to lethal control elsewhere^18^. Public opinion is similarly polarized, and contested views of dingoes mean that they are both revered and persecuted, and public interest has inevitably led to dingo management becoming politicised and highly emotive^19,20^.

To many Australians, the dingo is of deep cultural importance, including a deep association with Indigenous culture spanning thousands of years^8,20^. The value of dingoes also extends to ecosystem health, with a growing body of evidence confirming their positive influence on native biodiversity and ecosystem functionality^21–25^. As a highly interactive, top-order predator, dingoes have a range of direct and indirect effects on the ecosystems in which they inhabit, and many studies report that their removal from ecosystems may have negative consequences over multiple trophic levels^23,26–28^. Although currently unlisted, the dingo was classified as Vulnerable by the IUCN Red List from 2004 to 2018 and the current population trend of ‘pure’ animals is in decline^18^, and it is generally accepted that the principal threat to the species is loss of genetic integrity through introgression with domestic and feral dogs^18^. Since 1788, the destruction of dingoes to protect livestock interests has been widespread, with culling associated with bounties on scalps and skins presenting a significant threat to the species^18^.

The iconic dingo population on K'gari-Fraser Island (hereafter K’gari) is reputed to be one of the last remaining reservoirs of genetic purity for the species^16^, although no research has been conducted to support this assertion, and the lack of a definitive test for purity would likely undermine any such efforts. Regardless, the K'gari population is particularly representative of the ecological, aesthetic and cultural value of the species and the challenges faced in contemporary situations of dingo and human co-occurrence. K’gari is the world’s largest sand island (1840 km^2^) is located off the south-eastern coast of Queensland, Australia and World Heritage listed since 1992. Whilst the timing and exact nature of the dingo’s introduction to the island is unknown, K’gari has been separated from the mainland following the Holocene sea level transgression (~ 6000 YBP)^29^. The resident dingo population is recognised for its ecological role as a contributor to regulating the island’s biodiversity^30^. K’gari’s Traditional Owners, the Butchulla People, have longstanding cultural and spiritual connection with wongari (dingoes) and facilitated assisted migration of dingoes from mainland Australia during pre-colonial times. In contemporary times, the K’gari dingo population is also a compelling tourist drawcard as the most publicly recognisable dingo population in Australia^31^.

Negative human-dingo interactions on K’gari arise primarily from human associated activities, such as habitat encroachment, the creation of unnatural food resources and direct or indirect encouragement of dingoes in order to cultivate an interaction with an infamous terrestrial predator. These negative interactions can range in severity from stolen food resources and damage to human property, through to displays of aggression and/or actual physical harm. The worst incident to date occurred in 2001 and resulted in the death of an 11-year-old boy^19,32,33^. The occurrence of these incidents has necessitated the implementation of management strategies that include non-lethal options, such as temporary area closures, strategic fencing and public education measures. In some instances, where dingoes are deemed to be high-risk to human safety, removal via lethal control occurs. Due to their distinct hierarchical social order, such methods have the potential for far reaching negative effects to the population’s viability^34^, although this partly depends on the scope and scale of selective removal activities.

The K’gari dingo population has been under heightened pressure since the island was heritage listed and will face continued threats as annual human visitation (currently approximately 400 000) is predicted to increase^30^. As such, these pressures will likely result in a commensurate increase in sought after interactions, alongside the resultant occurrence of negative human-dingo interactions, albeit at a low background rate relative to the nature and scale of human visitation. Management challenges are becoming evident in balancing the conservation requirements for the island’s dingo population, whilst ensuring human safety.

The K’gari-Fraser Island population is small (~ 70–173 individuals^35,36^), isolated from mainland populations and subject to historical and ongoing culling events. Given the confluence of these factors, we hypothesise that the K’gari dingo population may be suffering negative genetic consequences, which may represent a conservation concern for the species. Therefore, knowledge of the population’s genetic status relative to continental populations is an essential first step in informing ongoing management. The quantification of genetic diversity of threatened species facilitates an interpretation of the manner in which diversity is distributed within and among populations^37,38^, leading to an understanding of factors such as gene flow, inbreeding, mutation, selection and genetic drift^38^. Knowledge of the population genetic structure of a species can provide insight into how a species may be affected by perturbations to these processes, or by historical factors such as founder effects or genetic bottlenecks^39,40^. Familiarity with the amount and distribution of genetic variability within a species will therefore increase the precision and effectiveness with which the main priorities for conservation management are identified and implemented, particularly for island populations, which may be more vulnerable to the adverse genetic consequences of isolation and small population size^39,41^.

Much of the current literature on dingo genetic research relates to the species’ taxonomic status and past radiations into and throughout Australia^9,10,12,15,42,43^, or to methods of determining levels of purity and hybridisation^42,44–46^. Whilst a handful of studies discuss genetic diversity and population genetic characteristics^46–48^, to date, only one study, with a relatively small sample size (*n* = 5) has included K'gari individuals in a discussion of population genetic parameters such as inbreeding and genetic diversity^49^. This paucity of genetic data will inevitably compromise effective conservation management of an iconic dingo population, which is isolated from the mainland on a World Heritage listed island and is of considerable public interest.

Based on the hypothesis that there may be conservation concerns associated with genetic factors relevant to small, island-bound populations, the primary objective of this study is to address the lack of population genetic data relating to the K'gari dingo population. Specifically, this research aims to compare genetic diversity measures with dingoes from the coastal regions adjacent to the island as well as regional districts up to 500 km inland, and to establish whether genetic drift has occurred. We will frame our results to explore conservation issues that may be associated with our population genetic data.

## Results

### Genetic diversity

A total of 99 alleles were observed across the 439 analysed dingo samples, with 33 alleles in the K’gari population (175 samples) and 94 in the mainland population (264 samples). While there were 66 private alleles in the mainland population, there were only five private K’gari alleles and a high degree of allelic fixation in this population. For example, two loci (WANV142 and FH3591) were monomorphic in K’gari dingoes but had six and eight alleles, respectively, in the mainland population. For locus FH2168, where 27 alleles were observed in the mainland dingoes, and only 5 alleles in the island population, with two of those alleles found within 97.6% of K’gari individuals.

All key genetic diversity measures were significantly lower in the K’gari population (Table 1), including the number of alleles per locus (*A; p* < *0.001, U* = 74.50), allelic richness (*A*_R_; *p* < *0.001, U* = 75.00) private allelic richness (*P*_*AR*_; *p* < *0.001, U* = *80.*00) and expected heterozygosity (*H*_*E*_*; p* < 0.05, *t* = 0.037). Despite the low allelic diversity for the K’gari dingo population, no statistically significant evidence was found to suggest a recent genetic bottleneck for either population (*p* > *0.05*). The fixation index was close to neutral for K’gari, and not significantly different (*p* > 0.05) than the low homozygosity excess observed in the mainland dingoes (Table 1). However, the effective population size (*N*_*e*_; Table 1) for K’gari dingoes was 24.7 (*p* < *0.05*; 95% C 14.0–45.8) compared to 103.8 for mainland dingoes (*p* < *0.05*; 95% CI 86.4–126.3).

**Table 1:** Summary of genetic measures for the K’gari and mainland dingo populations. *n*, number of dingoes sampled per population; *A,* mean number of alleles per locus; *A*_*R*,_ allelic richness; *P*_*AR*_, private allelic richness; *H*_*O*_, mean observed heterozygosity; *H*_*E*_, mean expected heterozygosity; *F*, fixation index; *N*_*e*_, effective population size, statistically significant difference (*, *p* < 0.05; **, *p* < 0.001) between K’gari and Mainland.

Population	*n*	*A*	*A*_*R*_	*P*_*AR*_	*H*_*O*_	*H*_*E*_	*F*	*N*_*e*_	*PhiPt*
K’gari	175	3.667*	3.61*	0.56*	0.271	0.288*	0.060	25.7	0.376**
Mainland	264	10.444	9.93	6.87	0.522	0.600	0.134	103.8	

### Genetic differentiation

There was evidence of genetic differentiation between the island and mainland population, with AMOVA indicating 38% of the variation due to differences among populations and a relatively high PhiPT value (Table 1; 0.376; *p* < 0.001*)*. Except for one individual in the K’gari population that clustered with the mainland individuals, the principal coordinates analysis demonstrated a clear difference in allelic composition between the two populations (Fig. 2). The genetic differentiation between the K’gari an mainland dingoes indicated in the PCoA analysis is also clearly demonstrated in the STRUCTURE analysis (Fig. 3; *K* = *2–4*) with 98% of K’gari individuals being characterised by one genetic cluster and 98% of mainland dingoes characterised by varying proportions of the two remaining genetic clusters (Fig. 3; *K* = 3). At *K* = 2, the K’gari dingo that clustered with the mainland population is clearly identified. The evidence of allelic fixation in the island population and genetic differentiation from the mainland population was clearly supported by the PCoA, AMOVA and STRUCTURE analysis.

## Discussion

Genetic variation provides a fundamental basis for evolutionary change and forms a significant factor in the ability of populations to adapt and evolve in response to extrinsic environmental pressures^50^. The magnitude and distribution of genetic variation within a species is formed externally by selective regimes resulting from climatic and ecological processes, as well as intrinsic factors such as breeding systems and life-history characteristics^39,51^. Hence, a species’ ability to maintain genetic diversity is often essential in ensuring its long-term persistence^38,52^. Species, or populations, that lack genetic diversity may have a heightened risk of extinction, due to lowered adaptive ability^38,39,53^.

Previous research has shown the Australian dingo to be a species with relatively low genetic diversity. Studies using codominant microsatellite markers, such as those used in this study, indicated that the genetic diversity of dingoes was markedly lower than for domestic dogs^47^. Similarly, results using maternally inherited mtDNA markers also demonstrated low genetic diversity, with only two unique haplotypes present, hence indicating the possibility of only one or two initial founder events in NE and/or NW Australia^10,43^. Although other research points to the possibility of multiple founder events^54^, the mtDNA evidence for low genetic diversity is also supported by work with paternally inherited Y-chromosome markers, which have demonstrated low diversity compared to SE Asian dogs^55^, alongside the detection of only two haplotypes in dingo populations vs 27 haplotypes that have been identified for domestic dogs^13^. While the exact number and geographic route of initial founder events remains under debate, cumulative evidence suggests that only a small number of founder events occurred, with only a small population of animals, and/or from a canid population that had undergone severe genetic bottlenecks, hence resulting in the establishment of the original dingo population in Australia with a relatively low level of genetic diversity^10,12,13,43,47^.

Genetic theory predicts, that under most scenarios, island populations will be less genetically diverse than their mainland counterparts^56^. This is usually a result of smaller population sizes at foundation, the effects of genetic bottlenecks, or genetic drift due to an absence of ongoing migration from more genetically diverse source populations^56^. As the first study to incorporate a large sample size of K’gari dingoes, our results confirm low genetic diversity for the K'gari population, with all diversity measures markedly lower than for the mainland dingoes from adjacent mainland Australia, despite the larger sampling area for the latter. A previous study incorporated a small sample size of the K’gari population (*n* = 5) and also found genetic diversity to be low^49^. These results are not surprising, as genetic erosion often occurs in geographically-isolated island fauna populations^57–59^, including for international taxa such as Arctic Island wolves (*Canis lupus arctos*)^60^ and for other threatened Australian native carnivores, such as the northern quoll (*Dasyurus hallucatus*)^61,62^. For example, in the study of northern quolls on Koolan Island, Western Australia, Spencer et al.^62^ found that despite a relatively high number of alleles present in the resident population, genetic diversity was lower than for mainland populations, and only contained 34% of the species’ allelic richness. Both Cardoso et al.^61^ and Spencer et al.^62^ conclude that for many island populations, low diversity results from random selection following a founder event from individuals that only represent a small proportion of the entire species diversity. The net effect is that island populations may be less likely than their mainland counterparts to persist in the long-term, due to a loss of evolutionary potential^38,39,61^. Although rigorously established demographic data for the K’gari dingo population is lacking, pilot studies have estimated either 104–197^36^ or 73–173^35^ dingoes to be present on the island, whilst another study estimated a population size of 76–171 in 2012^63^ indicating a small population according to island population genetic theory^38,56^. As such, there is strong evidence from our results that the K'gari-dingo population is suffering from the loss of genetic diversity that is typical of small, island populations. Although we found no compelling evidence of recent genetic bottlenecks, it is probable that the initial founder population represented a limited genetic diversity of the wider species, which when coupled with negligible gene flow from mainland dingoes, has led to further erosion of the population’s genetic diversity.

Another potential consequence of small, island-bound populations is the potential for increased inbreeding^56,64^. Cairns et al.^49^ found extremely high levels of inbreeding present in the K’gari dingo population (*F*_*IS*_ = 0.700^49^). However, those figures should be treated with caution given the low sample size (*n* = 5), and the potential inclusion of 2–3 siblings (pers. Comm; Queensland Parks and Wildlife). In our study, values for the fixation index were effectively neutral for the K’gari population, and thus we found limited evidence of inbreeding.

Effective population size (alongside census size) is an important consideration for small and isolated populations, and generally positively correlated with a population’s likelihood of persisting and ability to evolve in a changing environment^65^. While there are multiple arguments for an exact minimum viable population (MVP) threshold for effective population size^66^, recent research suggests ≥ 100/1000 is more realistic than the previous ≥ 50/500 rule^67^, with the respective figures relating to the likelihood of short and long-term persistence. The K’gari population has an effective population size of 25.7, which is considerably lower than both reported thresholds for short-term persistence (≥ 100/1000 and ≥ 50/500), and hence represents a significant conservation concern. While a lack of population size data (and difference in sampling area) for the mainland dingoes means that limited census vs effective population size comparisons can be made from our results, the fact that the K’gari *N*_*e*_ of 25.7 is considerably lower than the effective population size calculated for the sampled mainland dingoes (*N*_*e*_ = 103.8), also supports our other evidence relating to decreased diversity in the K’gari population. For example, despite the lack of excess heterozygosity indicating no recent population bottleneck, this supports our theory of significant levels of genetic erosion most likely caused by drift following founder effects and/or a historical bottleneck. Although fluctuations in population size on the island have not been monitored, initial founder effects (have no doubt played a role in the reduced genetic diversity relative to adjacent mainland populations and there is anecdotal evidence of higher population size on the island prior to the 1920s^68^. While the timing of the arrival of dingoes in Australia is still under debate and may be anywhere between 3500 and 18,000 YBP^10–12,43^, it is uncertain whether the founder population existed prior to the island’s separation from the mainland (~ 6000 YBP^29^), or whether the founder population was a result of repeated, small-scale, assisted migration events associated with the K’gari’s traditional owners^68^. Additionally, K’gari has not been immune to the post-colonial persecution of dingoes that occurs throughout Australia, which is best exemplified by the bounty system on dingo scalps that still exists under many jurisdictions in non-protected estate^17^. For instance, prior to K’gari being gazetted as a national park and achieving World Heritage status, there is documentation of a large-scale culling event (~ 100 individuals) initiated by one person to take advantage of the scalp bounty in the early 1900s^68^. Selective removal of problematic dingoes still occurs on the island, with 110 dingoes euthanised in the period 2001–2013^63^, which is a relatively high proportion given the low overall population size.

**Figure 1:**
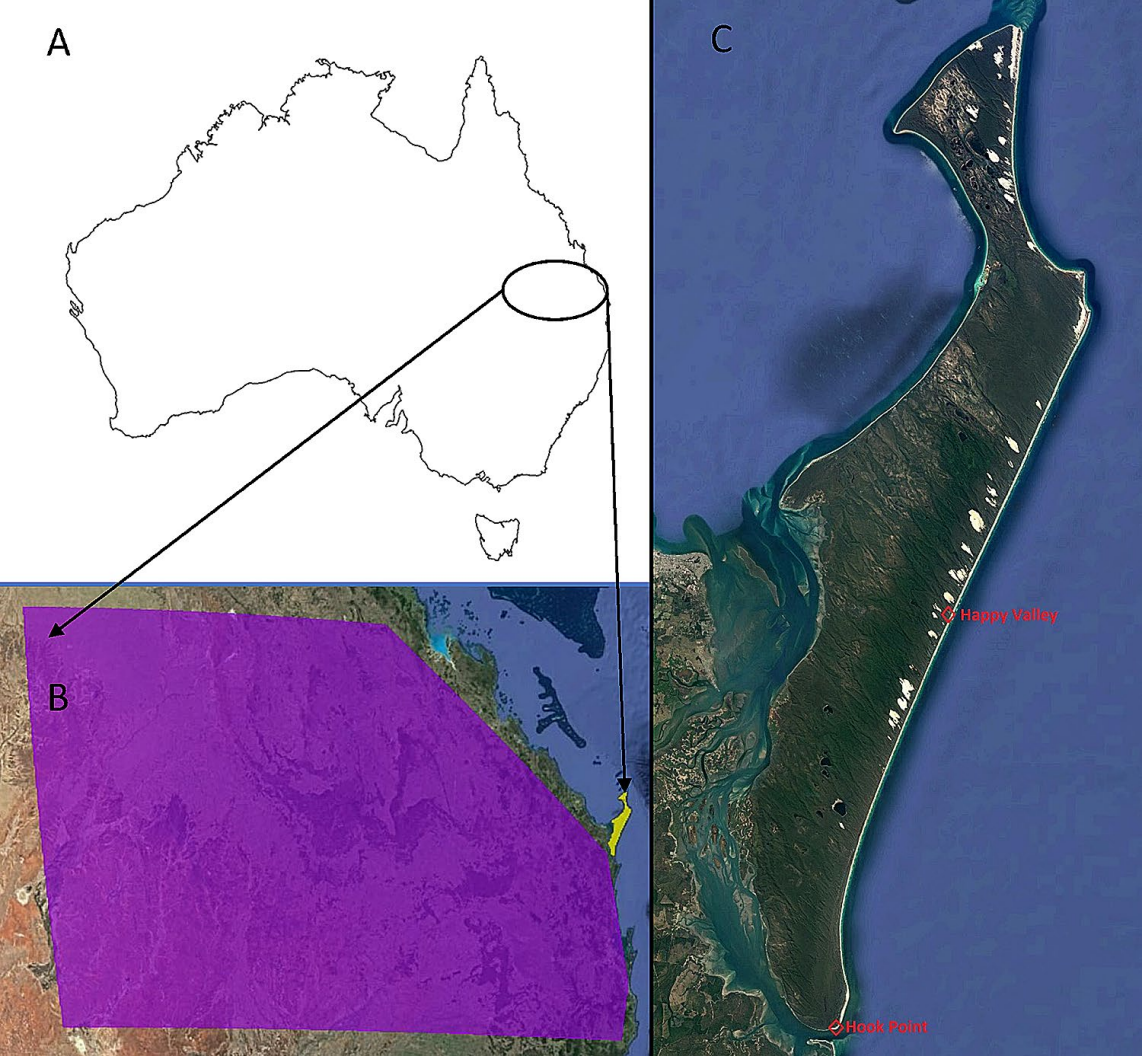
(**A**) The general study region in Australia (**B**) the mainland sampling region (shaded purple) adjacent to K’gari (shaded yellow), (**C**) K’gari-Fraser Island, with Hook Point and Happy Valley indicated. Maps were produced using Google Earth satellite imagery (Map data: Maxar Technologies) and ESRI ArcGIS software, Version 10.3 (http://www.esri.com/software/arcgis/arcgis-for-desktop).

**Figure 2:**
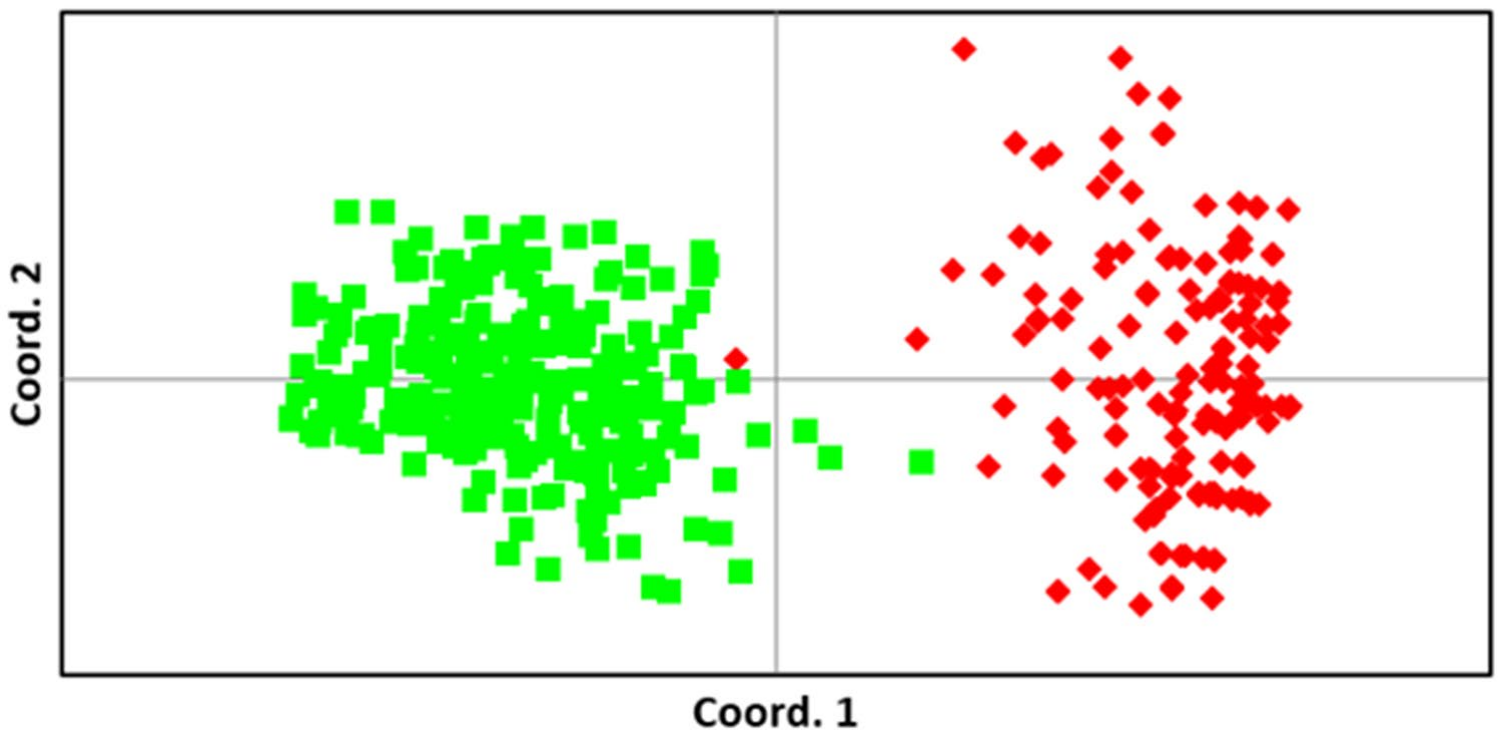
Principal coordinates analysis (PCoA) using genetic distance matrices. Individuals from the K’gari (red) and Mainland (green) populations are indicated. Coordinate axes 1 and 2 account for 24.75% and 5.30% of the variation in the data.

**Figure 3:**
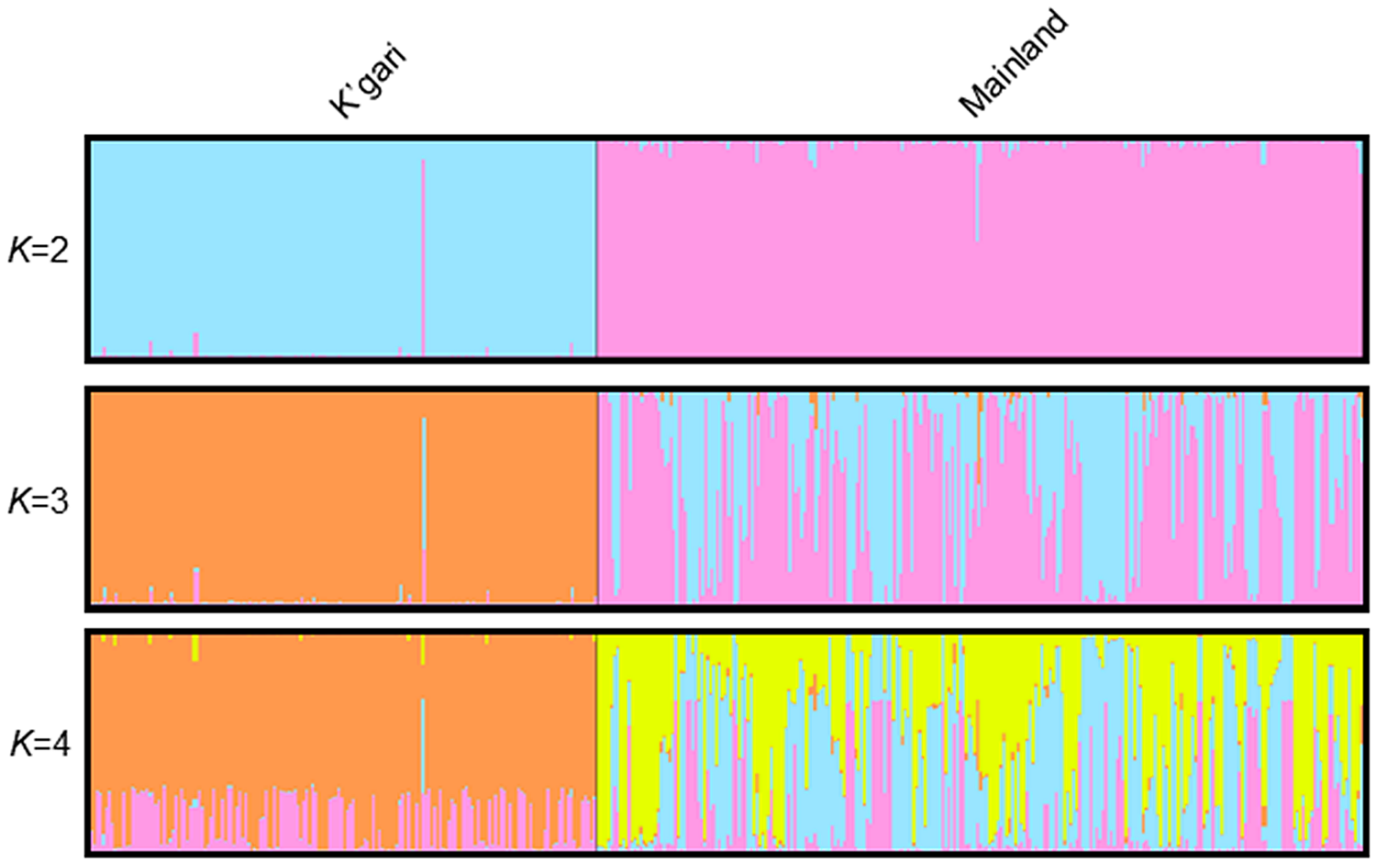
STRUCTURE admixture barplots for values of K = 2–4 showing the genetic relationships between the K’gari and mainland dingo populations. *K* = 3 was selected as the best estimate of the number of genetic clusters following implementation of the Evanno method.

Alongside reduced diversity, island populations often diverge genetically from their mainland founder populations^56^. In our study there was evidence of genetic differentiation (*PhiPt* = 0.376) between the K’gari population and the sampled mainland dingoes, which were sourced from adjacent regions in Queensland (Fig. 1). Both the PCoA and STRUCTURE analyses (Figs. 2 and 3) demonstrate a clear delineation between these two dingo populations based on allelic composition, which generally implies genetic isolation. There is evidence of at least one potential occurrence of migration from the mainland (whether assisted or ‘natural’) as demonstrated by the one K’gari dingo that appears to have a ‘mainland’ genetic signature (Fig. 2). This individual was sampled from Happy Valley (Fig. 1C) on the eastern side of K’gari in 2002 and may represent a rare immigration event. The southern tip of K’gari (Hook Point; Fig. 1C) is only separated from the mainland by ~ 1 km and there is anecdotal evidence to suggest that animals such as feral pigs (*Sus scrofa*) and domestic dogs (*Canis familiaris*) occasionally traverse this distance. Assisted (illegal) migration is also a possibility, particularly as this was in the year immediately following the killing of a human child by a dingo, when public opinion regarding dingo management was particularly fraught, and 31 dingoes were euthanised. Despite the possibility of rare immigration events, domestic dogs have been legally excluded from K’gari since 1981 and there is negligible evidence of significant levels of natural gene flow from the mainland. As such, genetic drift has likely occurred, and as a result the K’gari population has become genetically distinct. Whilst this could be interpreted as a highly unique population, it is important to consider that this genetic differentiation is mostly due to reduced genetic diversity and allelic fixation in the K’gari population because of founder effects in a small population (census and effective) following a historical genetic bottleneck.

Although assisted migration occurred prior to the colonial era (~ 1840) during due to the Butchulla People’s close links with dingoes, the island has been effectively isolated from the mainland following separation with the increase in sea levels during the Holocene transgression^29^. In combination with complex, hierarchical pack structures and breeding patterns^69^ that means that generally only alpha pairs yield offspring that survive past infancy, the genetic consequences have become more pronounced. This is clearly evidenced in the low effective population size, low genetic diversity and high levels of allelic fixation relative to adjacent mainland dingoes. Thus, while we found no evidence of inbreeding there is cumulative alternative evidence to suggest that the current population is suffering negative genetic effects, which may ultimately compromise its evolutionary potential and thereby place the population at greater risk of stochastic extinction.

### Management implications and further research

The K’gari dingo population is typical of small, isolated island populations, as exemplified by low genetic diversity, low effective population size, and differentiation from adjacent mainland dingoes. This is likely a product of low initial genetic diversity following an initial founder event and flow-on effects, such as genetic drift, which has been exacerbated by an absence of substantial immigration from outside sources. In total, 110 dingoes were selectively removed on K'gari in the period 2001–2013^63^ due mostly to negative human-dingo interactions. The most significant event occurred in 2001, when 31 dingoes were killed following the death of a young child from a dingo attack, plus 1 additional dingo in an unrelated incident^19,70^. There are also many other forms of mortality of K’gari dingoes including attacks by other dingoes and vehicle strike. Given that the high rates of human visitation are likely to continue, human associated forms of mortality will be an ongoing management issue. The authorities responsible for managing K’gari published a Fraser Island Dingo Conservation and Risk Management Strategy (FIDCRMS) which states the “conservation and preservation of a sustainable wild dingo population” as a priority, whilst highlighting that effective management needs to be supported by research that allows the “timely identification of concerning trends regarding impacts to dingo conservation”^71^.

Whilst our use of neutral markers precludes the direct measurement of genetic ‘health’, we find clear evidence of reduced genetic diversity relative to adjacent mainland populations and a low effective population size. It is well established that both of these factors result in lowered adaptive capacity and increased extinction risk, both of which represent conservation concerns. As such, determining management priorities for the K’gari dingo population will need to be supported by further research. Although the data from this study provides an invaluable baseline, there are precedents of the need for ongoing genetic monitoring, in association with demographic monitoring to maximize the potential for the persistence of small, genetically compromised island populations^57,61,62^. Without long-term genetic and demographic monitoring, it is unclear whether increasing human activity will lead to a reduction in population size and further erosion of genetic diversity. Clarification of these points is crucial, as repeated reductions in population size may give rise to numerous demographic and genetic factors which may compromise persistence, and ultimately lead to localised extinction^38^.

To support the management priorities outlined in the FIDCRMS, we recommend that further genetic analyses be undertaken. Given ongoing advancements in molecular genetic technologies, it is advised that future studies utilise sequencing platforms that will have a higher degree of sensitivity and data resolution, including the potential inclusion of approaches that facilitate the monitoring of genetic markers associated with fitness. This will provide additional highly informative molecular resources for future dingo research as well as clarity on levels of inbreeding and the presence of deleterious allele accumulation in the population (genetic health). It is also highly recommended that an ongoing genetic and demographic monitoring program is established to more fully understand chronological trends and responses to extrinsic impacts that may compromise the persistence of a robust population.

## Methods

### Sample source and experimental design

One hundred and seventy-five frozen, viable K’gari dingo tissue samples were provided by Queensland Parks and Wildlife Service (QPWS). These samples were collected between 1999 and 2014 from all areas of K'gari during routine QPWS activities (Fig. 1). Genetic data obtained from tissue from 264 dingoes collected between 2003 and 2009 from a larger geographic area in adjacent southern and central mainland Queensland were used for comparison to the K’gari samples (Fig. 1) and analysed with identical PCR and genotyping methods. As no physical barriers to gene flow (e.g. dingo fence) exist in the mainland study region, they can be considered a contiguous population.

### Laboratory methods

Genomic DNA was extracted from the K’gari samples using a DNeasy Blood and Tissue Kit (Qiagen, Hilden, Germany) according to the manufacturer’s instructions. Mainland samples were extracted using the method of Ivanova et al.^72^. Nine polymorphic microsatellite loci (FH2168, FH3591, WanV142, FH3413, Ren195, FH2537, FH3278, Ren47D, Ren229; Supplementary Table S1), isolated from the dog genome, but used in previous dingo population genetic studies^46^) were utilised to assess population genetic variation by employing PCR and capillary electrophoresis. Forward and reverse primers for each microsatellite loci were used in a 10 μL PCR, consisting of 5 μL Qiagen Multiplex PCR solution (Qiagen Inc. Valencia, CA, USA), 1 μL Qiagen Q-Solution, 2 μL DNA, 0.2 μm of each primer and DNase-/RNase-free water. Multiplex amplification was performed using an Eppendorf Mastercycler (Hamburg, Germany) with cycling conditions as follows: initial denaturation at 95 °C for 15 min, followed by 35 cycles of 30 s at 94 °C, 90 s at 58 °C and 60 s at 72 °C, with a 30 min final extension at 60 °C. PCR products were separated by capillary electrophoresis on an AB 3730 Genetic Analyser (Applied Biosystems) and scored relative to an internal lane standard (GS-500 LIZ; Applied Biosystems) using GENEMARKER v. 2.4.0 (SoftGenetics LLC, PA, USA) and checked twice by two independent researchers. Consistent amplification was observed through visualisation using agarose gel electrophoresis and clear electrophoretic signatures were observed following capillary electrophoresis. Individual samples that failed to yield peaks were amplified and genotyped for a second time.

Quality control of the allelic data was performed using a range of analyses^73,74^. The potential for null alleles was determined using MICRO-CHECKER v2.2.3^75^ and ML-NULLFREQ^76^, based on 1000 bootstraps. GenAlEx was used to estimate deviation from Hardy–Weinberg equilibrium for each locus^73,77^, and an assessment of linkage dis-equilibrium among pairs of loci was undertaken with FSTAT v2.9.3.2^78^. Large allele dropout and stuttering potential was assessed with MICRO-CHECKER v2.2.3^75^, based on 1000 bootstraps and a 95% confidence interval. Evidence of linkage disequilibrium was detected in one loci (FH3591). Although eight of the loci deviated significantly from HWE after Bonferroni correction, the deviation was unsurprising given that isolated, island populations are expected to be affected by selection pressures^73^. MICRO-CHECKER and ML-NULLFREQ estimated the average potential null allele frequency to be 0.13 ± 0.02, and 0.12 ± 0.02 across all loci, respectively, which can potentially lead to underestimation of diversity and differentiation parameters. Additionally, given the fact that loci from wild populations affected by bottlenecks, inbreeding, genetic drift, founder effects and natural selection are likely to deviate from HWE^79^, it is unlikely that these deviations will significantly impact subsequent genetic analyses^73^.

### Genetic diversity

GenAlEx v. 6.503^77^ (Peakall and Smouse 2012) was used to generate allelic frequencies, and to calculate a range of population genetic parameters^74^ including the mean number of alleles per locus (*A*), observed and expected heterozygosity (*H*_*O*_ and *H*_*E*_), and the fixation index (*F*) as an estimate of past inbreeding. Allelic richness (*A*_*R*_) and private allelic richness (*P*_*AR*_) per population were calculated in HP-RARE^80^, using rarefaction to account for unequal sample size. Differences between the diversity and inbreeding measures for the K’gari and mainland populations were analysed using independent sample T-tests (normal data) or Mann–Whitney U-tests (skewed data) using SPSS software (SPSS Inc. Chicago).

To detect the likelihood of recent bottlenecks, the program BOTTLENECK was used to test for mutation-drift equilibrium^81^. The observed level of heterozygosity was compared to the expected HetEQ using the intermediate two-phased model (TPM), due to its suitability for microsatellite data^81,82^. The TPM was applied with 95% of the mutations following a two-phase mutation pattern and a variance among multiple steps of 12^83^, with deviations from equilibrium determined using Wilcoxon’s Sign Rank tests, which are most suitable in instances where less than 20 loci are used^81^. Effective population size (*N*_*e*_) was estimated assuming a closed population with discrete generations and random variance in reproductive success using the program NeEstimator v2.01^84^. The linkage disequilibrium method (LD) was used with the random mating model and results were reported for *P*_*crit*_ 0.02 which is recommended for greater precision with microsatellite markers^84,85^.

### Population genetic structure

Genetic distance^86^ was calculated using GenAlEx v. 6.503^77^, to establish genetic relationships within and among populations. The average pair-wise genetic differentiation between populations (*PhiPt*) was calculated using multi-locus comparisons based on 999 permutations. The partitioning of genetic variance within and among populations was determined using AMOVA^87^ with GENALEX version 6.503^77^.

A principle coordinates analysis (PCoA) was conducted in GENALEX version 6.503^77^ using standardised genetic distance measures and we examined population structure further using the Bayesian clustering algorithm implemented in STRUCTURE v2.3.4^88^. We used an admixture model with correlated allele frequencies and 10 independent runs for each value of *K* (number of clusters)^73,74^ between 2 and 6, employing a burn-in of 100 000 followed by 500 000 Markov Chain Monte Carlo (MCMC) steps for each run. The geographic location of samples was not used in the clustering analysis. Results across each run were summarised to determine the optimal value of *K* using the Evanno^89^ method, as implemented in STRUCTURE HARVESTER web v0.6.93^90^, processed using CLUMPP v1.1.2^91^ to determine the optimal alignment of the ten iterations and finally plotted with DISTRUCT v1.1^92^.

## Supplementary Information

Supplementary Information
