# Supplementary material for: Conservation concerns associated with low genetic diversity for K’gari–Fraser Island dingoes
Source: Sci Rep. 2021 May 4;11:9503. doi: 10.1038/s41598-021-89056-z (PMC8097078; doi:10.1038/s41598-021-89056-z)
Supplement: Supplementary file 1 — Supplementary Information [file 41598_2021_89056_MOESM1_ESM.pdf]

Title:

Conservation concerns associated with low genetic diversity for K'gari-Fraser island dingoes within a UNESCO Biosphere Reserve

Authors:

Conroy GC[1*], Lamont RW[1], Bridges L[1], Stephens D[2], Wardell-Johnson, A[3], Ogbourne SM[1]

Supplementary Table S1: Details of nine microsatellite loci utilised for genetic analyses. Primer sequence (5'-3'); *PIC* Polymorphic information content; N_A number of alleles; H_E expected heterozygosity; H_O observed heterozygosity.

Primer Name	Source	Forward Sequence	Reverse Sequence	PIC	N_A	H_E	H_O
FH2168	Francisco et al 1996[1]	GCA AAT TAC TTA CTT CAC TAT GCC	TTG CAA GAC TTC AAC ATG GC	0.852	27	0.700	0.668
FH2537	Guyon et al 2003[2]	AAA AAG TGT AGA GCT TTC TCC AAA	ATT GAG ACC CAA GAC TGT TAG TG	0.749	11	0.770	0.708
FH3278	Lingaas et al 2003[3]	CTG CTC TTT GTA ACC CAT GC	AAT GCC TAC CAG GTG AAG G	0.758	9	0.693	0.643
FH3413	Guyon et al 2003[2]	AGA GTT GAA AGG TTG AAA ATG G	TGT GGT CAC AAG ACT TTA GCC	0.778	17	0.533	0.449
FH3591	Guyon et al 2003[2]	AAC TGT CAC ACA ATG TTA CAT CC	TCA TTA CTA TGC AAA ACT TCA AGG	0.076	8	0.062	0.057
Ren195	Ostrander et al 1993[4]	GCT TTC CCA TTG TGT CCT CA	TGA TTG ATG CCC TTT CAA CA	0.185	6	0.159	0.125
Ren229	Breen et al 2001[5]	ACT GAC TGA TGT CCT GTG CG	ATC TAC CCT GCA ACC CTG TG	0.754	8	0.619	0.517
Ren47D	Jouquand et al 2000[6]	GGC ACT TGA GCT CTA ATC CTA	TGC TAA TGA ATC CAC AGA ATG	0.263	7	0.213	0.179
WanV142	Ostrander et al 1993[4]	AAG CAG ATC CTA GAG CAG CA	CCC CAC AGT TTA GAA ATA TCT GC	0.522	6	0.249	0.225
			**MEAN:**	0.549	11	0.444	0.397

References

1 Francisco, L., Langsten, A., Mellersh, C., Neal, C. & Ostrander, E. A class of highly polymorphic tetranucleotide repeats for canine genetic mapping. *Mammalian Genome* **7**, 359-362 (1996).

2 Guyon, R. *et al.* A 1-Mb resolution radiation hybrid map of the canine genome. *Proceedings of the National Academy of Sciences* **100**, 5296-5301 (2003).

3 Lingaas, F. *et al.* A mutation in the canine BHD gene is associated with hereditary multifocal renal cystadenocarcinoma and nodular dermatofibrosis in the German Shepherd dog. *Human molecular genetics* **12**, 3043-3053 (2003).

4 Ostrander, E. A., Sprague, G. F. & Rine, J. Identification and characterization of dinucleotide repeat (CA) n markers for genetic mapping in dog. *Genomics* **16**, 207-213 (1993).

5 Breen, M. *et al.* Chromosome-specific single-locus FISH probes allow anchorage of an 1800-marker integrated radiation-hybrid/linkage map of the domestic dog genome to all chromosomes. *Genome Research* **11**, 1784-1795 (2001).

6 Jouquand, S. *et al.* Identification and characterization of a set of 100 tri-and dinucleotide microsatellites in the canine genome. *Animal Genetics* **31**, 266-272 (2000).
